# Non-Contact Evaluation of Pigs’ Body Temperature Incorporating Environmental Factors

**DOI:** 10.3390/s20154282

**Published:** 2020-07-31

**Authors:** Guifeng Jia, Wei Li, Junyu Meng, Hequn Tan, Yaoze Feng

**Affiliations:** 1 College of Engineering, Huazhong Agricultural University, Wuhan 430070, China; guifeng@mail.hzau.edu.cn (G.J.); lw_liwei@webmail.hzau.edu.cn (W.L.); Junyum@webmail.hzau.edu.cn (J.M.); thq@mail.hzau.edu.cn (H.T.); 2 Key Laboratory of Agricultural Equipment in Mid-lower Yangtze River, Ministry of Agriculture, Wuhan 430070, China

**Keywords:** infrared imaging, pigs, temperature prediction model, support vector regression

## Abstract

Internal body temperature is the gold standard for the fever of pigs, however non-contact infrared imaging technology (IRT) can only measure the skin temperature of regions of interest (ROI). Therefore, using IRT to detect the internal body temperature should be based on a correlation model between the ROI temperature and the internal temperature. When heat exchange between the ROI and the surroundings makes the ROI temperature more correlated with the environment, merely depending on the ROI to predict the internal temperature is unreliable. To ensure a high prediction accuracy, this paper investigated the influence of air temperature and humidity on ROI temperature, then built a prediction model incorporating them. The animal test includes 18 swine. IRT was employed to collect the temperatures of the backside, eye, vulva, and ear root ROIs; meanwhile, the air temperature and humidity were recorded. Body temperature prediction models incorporating environmental factors and the ROI temperature were constructed based on Back Propagate Neural Net (BPNN), Random Forest (RF), and Support Vector Regression (SVR). All three models yielded better results regarding the maximum error, minimum error, and mean square error (MSE) when the environmental factors were considered. When environmental factors were incorporated, SVR produced the best outcome, with the maximum error at 0.478 °C, the minimum error at 0.124 °C, and the MSE at 0.159 °C. The result demonstrated the accuracy and applicability of SVR as a prediction model of pigs′ internal body temperature.

## 1. Introduction

The pig livestock sector is a main component of Chinese animal husbandry, and the pig industry faces many challenges. For instance, pigs are constantly threatened by infectious diseases, which will cause respiratory, digestive, or reproductive disorders, even leading to death, making the industry vulnerable and less efficient [1]. The recent outbreak of African Swine Fever was a living example of how much cost infectious diseases can cause [2]. With the trend of swine breeding and upbringing developing towards a large-scale and digitalized style [3], the early detection and prevention of swine epidemics has become a core issue for the swine industry. Fever is a signature of many infectious diseases, such as African Swine Fever (ASF), Pseudorabies (PR), Aftosa, and Swine Plague [4]. The early symptoms of these diseases might appear to be a body temperature rise of up to 41–43 °C [5]. Therefore, monitoring the body temperature of swine individuals enables the early detection of and quick response to epidemics.

In swine fever diagnosis, internal body temperature is the gold standard [6] and is normally measured via a mercurial thermometer. However, a mercurial thermometer requires invasive and time-consuming steps for acquiring the internal temperature from the rectum. Such invasive practice increases the possibility of cross-infection and is highly infeasible in large-scale farms. Infrared Thermography (IRT) is a non-contact way to measure surface temperature. IRT can detect surface temperature without invasive practices and incurring negative responses from the animal [7]. There are two IRT categories: active thermography and passive thermography. Passive-type IRT directly scans the heat radiance intensity and translates that into temperature, while active IRT relies on an external radiance source to distinguish the temperature difference between the object and background [8]. Active IRT is often applied to a material surface and internal nondestructive testing. The interior of the living pig can be regarded as a heat source, whose surface temperature dramatically contrasts with the surroundings. What is more, environmental radiance sources will influence the heat exchange between the pig‘s internal body and skin and stimulate the pig′s physiological response to heat, which may lower the accuracy of heat evaluation and harm animal wealfare. Therefore, passive IRT is more appropriate for animal body temperature evaluation. Wide applications of passive IRT include inflammation detection [9,10], ovulation monitoring [11,12], abnormal behavior identification [13], and growth evaluation [14]. However, IRT can only monitor the skin temperature of swine, which cannot directly provide evidence for fever diagnosis. The relationship between the skin temperature and internal temperature of swine remains undetermined. Therefore, in order to apply IRT to fever diagnosis, modeling the correlation between skin temperature and internal temperature becomes inevitable. Previous research has shown that the “thermal windows” of pigs, such as the sulcus auriculae posterior [15], eye [16], and vulva [11,12], are highly correlated with the surface temperature and internal temperature of swine. Such regions are usually referred to as regions of interest (ROI). According to the mechanism discovered by previous works, a quantitative model between ROI temperature and internal temperature can be constructed [17,18].

In reality, the surrounding temperatures in pig farms are mercurial, which is a result of multiple dynamic heat exchange methods, such as heat conduction and convection, between the skin of the pig and the surroundings [19,20]. Under this condition, skin temperature is not only subject to changes from the internal temperature, but is also influenced by the environment [21]. To make things more complicated, humidity can also have an influence on pigs’ skin temperature [22]. Therefore, merely relying on ROI skin temperature to infer internal temperature is unreliable, and will not likely provide cogent evidence for fever diagnosis [17]. Despite the fact that environment indicators play an unignorable role in influencing ROI skin temperature, the influence of ambient temperature and humidity on body temperature prediction has not been considered in current studies, which is a great limitation in improving the accuracy of body temperature measurements [23]. Therefore, in order to improve internal temperature detection via IRT, this paper incorporates environmental indicators and key ROI skin temperatures to formulate a model that measures the internal temperature. After the construction, the model was then evaluated and verified. Given that the validation method is accepted, IRT non-contact skin temperature detection can be applied on large-scale pig farms for fever diagnosis. An IRT inspection robot can be designed for large-scale pig farms, utilizing automation, remote communication, and online robot monitoring technologies to collect infrared images from above and behind the pigs and then apply object recognition techniques on the images to automatically detect the ROIs of the backside, the ear root, and the vulva, while recording the environmental humidity and air temperature. With all that, a model can predict the internal temperature of a pig and further infer fever or other health conditions. With all that information uploaded to an Internet of Things (IoT) system, veterinarians in the farm can get quick notice and then execute a quick response. Generally, the temperature prediction model introduced in this paper contributes to automation and mechanization for large-scale pig farm disease prevention and control.

## 2. Materials and Methods

The live animal experiment was performed in a semi-closed pig farm located in Linquan Cunty, Anhui Province, from 19th to 24th August, 2018. The experiment was conducted on 18 non-pregnant swine in fence rising mode. The swine have 2–3 periods of piglet delivery. The skin temperature was taken by the Fluke Ti-300 hand type infrared thermometer, whose resolution is 240 × 180 pixels, and its vision view angle is 24 °H × 17 °V. The thermometer has a precision of 2% and a sensitivity of 50 mK, and carries a laser distance sensor whose accuracy is 0.01 m. The laser sensor was used for distance measurement and automatic focusing. The Fluke Ti-300 can shoot infrared images and visible light images at the same time. The internal temperatures of swine were recorded using an animal-purposed mercurial thermometer. The air temperature and relative humidity were measured by the Victor VC231 environment gauge. The precision for air temperature is ±0.3 °C, and for relative humidity it is ±2%. It can tell a 0.1 °C difference for temperature and a 0.1% difference for relative humidity.

The swine were tested twice a day. The first test was arranged from 8 to 10 in the morning, and the second was from 16 to 18 in the afternoon. The ROI infrared images were taken before performing a rectum internal temperature measurement, so that the stress reaction invoked by the rectum insertion may not influence the skin temperature in the images. The ROI regions were the backside, ear root, eye, and vulva. The backside region was large. For the sake of consistency for the backside area, a square area of 5 × 5 cm^2^ was marked at a position 6 cm away from the center line of the swine′s last rib. The area was flat and recognizable, and therefore was determined as the backside ROI. A mercurial thermometer was inserted into the rectum after being sterilized by a 70% concentration alcohol to prevent cross infection. After the reading became stable, the air temperature and rectum temperature were both recorded. The IRT temperature range was set at −20–80 °C, and the emissivity was 0.97 [24,25]. The images were taken 0.3 m away from the ROI. A total of 216 data samples were obtained from the animal experiment.

**Figure 1:**
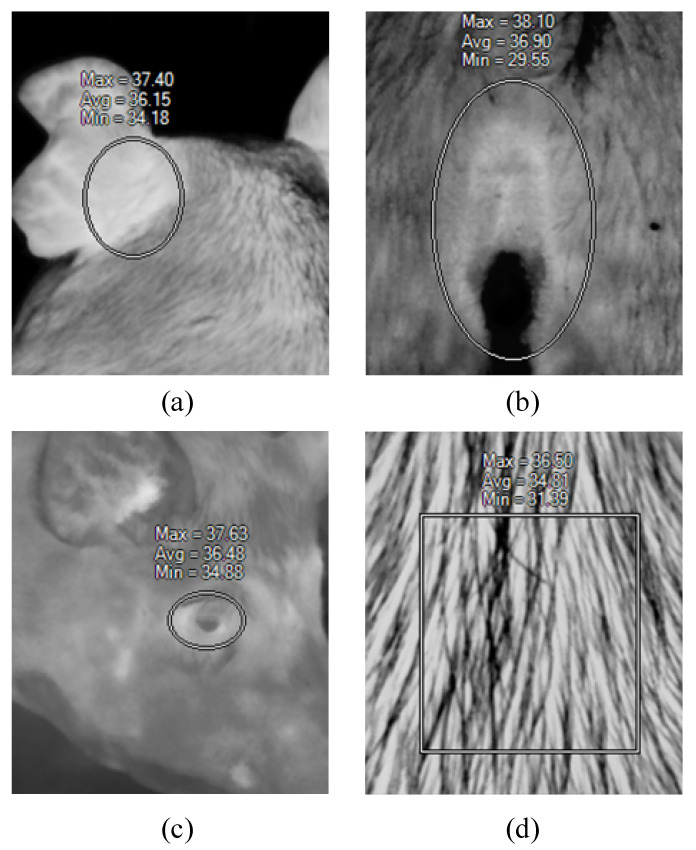
Region of interest (ROI) region definition in infrared image, (**a**) ear root ROI, (**b**) vulva ROI, (**c**) eyes ROI, (**d**) backside ROI. Note: the thermal images are displayed in a grayscale fashion.

The infrared images were processed using SmartView (Fluke Co, Ltd., Everett) software. The ROI in the images were selected using a rectangular or oval shape selection tool. Ear root is the position at the back and conjuncture with the head [26]. The location is shown in Figure 1a. The ROI for the eye and vulva are easy to identify in infrared images, and both were taken using the oval shape tool, as shown in Figure 1b, c. The backside ROI was compassed using rectangular shape tool, as in Figure 1d. SmartView automatically calculates the highest, lowest, and average temperature in the selected region. The ear root, eye, and vulva are thermal windows for swine, where plentiful blood vessels have developed subcutaneously in those regions. For this reason, the thermal window has a higher temperature in contrast to its surrounding skin areas [27]. Therefore, the ROI highest temperature was defined inside of this area. The lowest temperature was decided on the peripheral area, or on the bulge (such as in Figure 1b, the vulva bulge area), or the hair (like in Figure 1d, the back skin). The uncertain nature of the lowest temperature makes the indicator inconsistent. Hence, the ROI region temperature is represented by the highest temperature.

## 3. Data Statistics and Internal Temperature Prediction Modeling

### 3.1. Data Statistics

Based on a live animal experiment, the surface temperatures of the eye, ear root, backside, and vulva were extracted from the IRT images, and they are denoted *T_eye_*, *T_ear_*, *T_back,_* and *T_vulva_* respectively. The environmental indicators are air temperature and relative humidity, which are described as *T_air_* and *RH_air_*. With the surface temperatures indicators counted in, one sample contains seven indicators in sum. The maximum, minimum, and mean of the internal temperature (rectal temperature) are 39.20, 37.50, and 38.41 °C, respectively. The correlation between the ROI temperatures and environmental factors are shown in Table 1.

Judging from Table 1, the ROI temperatures of the backside, eye, ear root, and vulva are lower than the internal temperature by 1.60, 1.46, 0.98, and 0.94 °C, respectively. The temperatures of the backside, ear, and ear root have Spearman correlations with *T_air_*, and the numbers are 0.52, 0.32, and 0.28, respectively, with a high significance (*p* < 0.01). Meanwhile, the temperature of the vulva has a Spearman correlation with *T_air_* at 0.13 (*p* > 0.05). This shows that the backside ROI temperature is susceptible to environmental factors, the eye and ear root are less influenced, and the vulva is the least affected. The temperatures of the backside and eye are significantly correlated with the relative moisture, reaching −0.21 and −0.25, respectively. In short, the air temperature and relative humidity will influence the ROI skin temperatures via convection, conduction, and other heat transfer mechanisms. This shows that the skin temperature is a result of complicated influences from environmental factors and the internal body temperature. Thence, the modeling of internal temperature prediction should include environmental impacts. Besides this, relative humidity is negatively correlated with air temperature, reaching −0.81 (*p* < 0.01). Such findings correspond to the scenario where the relative humidity declines while the temperature rises in semi-open pig farms during summer.

### 3.2. Construction of Body Temperature Prediction Model and Evaluation Methodology

**Figure 2:**
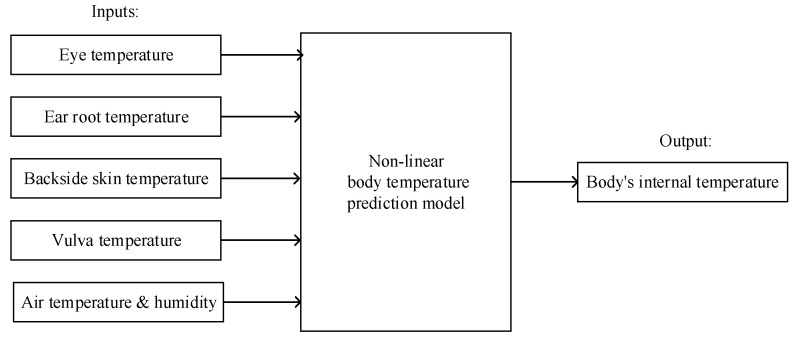
The inputs and output of the prediction model incorporating environmental factors.

Since pigs′ internal body temperature and ROI temperatures of the eye, ear root, and vulva are non-linearly correlated [17], the prediction model is based on a non-linear base model. In the application of nonlinear prediction, it is theoretically possible that machine learning demonstrates quicker and more accurate learning, broader generalization, and more stable performance than traditional approximation models. These machine learning algorithms include Support Vector Machine (SVM) [28,29], Random Forest (RF) [30,31], and Back Propagation Neural Network (BPNN) [32,33], and they are widely applied in practical problems involving pattern recognition and regression in various fields. Henceforth, SVR, RF, and BPNN are employed to predict body temperature and are evaluated individually. Meanwhile, in order to reflect the influence from environmental factors within our models, our models were constructed on datasets with and without environmental factors. When environmental factors were incorporated, the inputs were the ROI temperatures of the eye, ear root, backside, and vulva; air temperature; and humidity. The unincorporated input only includes the ROI temperatures. The inputs and outputs of the body temperature prediction model incorporating environmental factors are illustrated in Figure 2.

To quantitatively evaluate the influence of environmental factors, Root Mean Square Error (RMSE), Mean Absolute Error (MAE), Maximum Absolute Error (MaxAE), and the correlation coefficient *r* between the prediction and ground-truth temperatures were computed and taken as the evaluation evidence. The RMSE, MAE, and MaxAE were computed according to Equation (1), Equation (2), and Equation (3), respectively, where *y_i_* denotes the predicted value, ${\hat{y}}_{i}$ denotes the ground-truth value, and *N* is the number of samples.

(1) $$RMSE=\sqrt{\frac{1}{N}\sum_{i=1}^{N}({\hat{y}}_{i}-y_{i}{)}^{2}}$$

(2) $$MAE=\frac{1}{N}\sum_{i=1}^{N}\left|{\hat{y}}_{i}-y_{i}\right|$$

(3) $$\max AE=\max\left(\left|{\hat{y}}_{i}-y_{i}\right|\right)$$

### 3.3. Non-Linear Body Temperature Prediction Model

#### 3.3.1. Body Temperature Prediction Model Based on SVR

The Support Vector Regression machine (SVR) is a supervised machine learning technique based on the Statistical Learning Theory and Structural Risk Minimization Principal. SVR transforms the low-dimensional non-linear problem into a high-dimensional linear problem through the chosen non-linear mapping function. The mapping function is defined as Equation (4) [34].

(4) $$f=\omega^{T}\cdot \phi (x)+b$$

where *ω* is the weight vector, *b* is the offset, and *Φ(x)* is a feature vector that maps *x* into a high-dimensional space. To minimize the error, the nonnegative slack variables *ξ_1_*, *ξ_2_*, and putative factor *C* were introduced. This can minimize Equation (5), thus obtaining the SVR model.

(5) $$\begin{array}{l} \min\frac{1}{2}{\left\|\omega \right\|}^{2}+C\sum_{i=1}^{n}\left(\xi_{i}{}^{+}+\xi_{i}{}^{-}\right)\\ s.t.\{\begin{array}{c} y_{i}-\omega \cdot \phi (x_{i})-b\leq \varepsilon +\xi_{i}{}^{+}\\ \omega \cdot \phi (x_{i})+b-y_{i}\leq \varepsilon +\xi_{i}{}^{-}\\ \xi_{i}{}^{-},\xi_{i}{}^{+}\geq 0 \end{array} \end{array}$$

Radial Basis Function (RBF) is decided on as core function to solve the SVR model. The precision and generalizability of SVR depends on the selection of putative factor *C*, kernel function *g*, and loss function *e*; therefore it requires an optimal set of *C*, *g*, and *e* as the input. For a single-objective optimization problem, Genetic Algorithm (GA) consistently demonstrates a desirable ability to search for the global optimum. GA mimics the evolutionary process; searches for the optimum value from the initial population; computes the fitness value for each individual; performs the selection of elites, crossovers, mutations; spawns the next generation; then repeats the process until the end condition is satisfied. To obtain the optimum *C*, *g*, and *e*, GA and SVR were combined to predict body temperature. The objective function is the reciprocal of RMSE. The Genetic Algorithm Support Vector Regression (GA-SVR) algorithm procedure is described below:

1. Hyper parameter setup; set population at 60, crossover rate as 0.6, and mutation rate at 0.1; the iteration number is 200.
2. Initialize population; encode *C*, *g*, and *e* using 8-bit binary sequence into genes; the range of *C* is [0.1, 1], the range of *g* is [0.2, 0.3], and the range of *e* is (0, 0.1].
3. Translate genetic sequence into *C*, *g*, and *e* parameters; train the SVR model and evaluate model using Leave One Out Cross Validation (LOOCV); compute the RMSE; and take reciprocal of RMSE as the fitness function, as shown in Equation (6):
  (6) $$f=\frac{N}{\sum_{i=1}^{N}{\left(y_{i}-\bar{y_{i}}\right)}^{2}}$$
4. Decide whether the ending condition is satisfied. If not, select elite individuals according to the roulette wheel selection principal and replicate elites, then perform cross over and mutation and return to step (3). If the ending condition is met, end the iteration.
5. Output the optimum parameters C, *g*, and *e* and corresponding MSE, end of algorithm.

**Figure 3:**
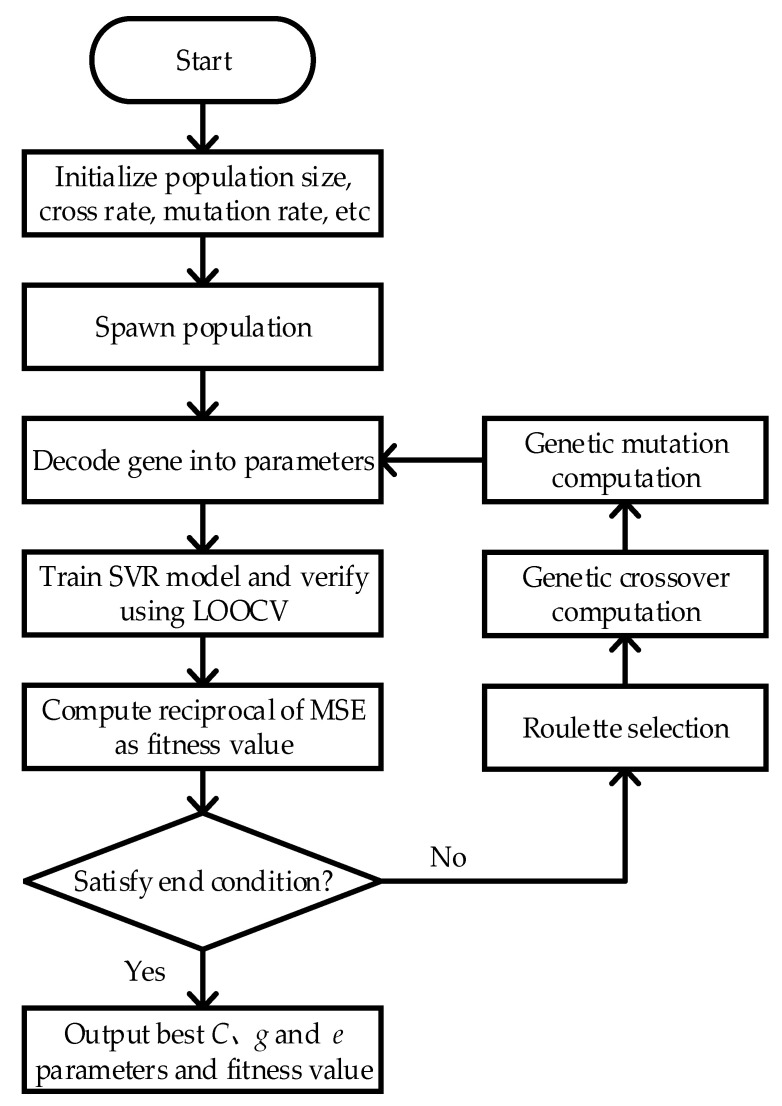
Flowchart of GA-SVR.

In short, the flowchart of GA-SVR is shown in Figure 3. The algorithm and objective function were implemented in MATLAB, where the SVR part is implemented in Libsvm [35].

#### 3.3.2. Body Temperature Prediction Model Based on RF

RF is a hybrid learning methodology combining a classification regression tree and decision tree, making the algorithm efficient and robust. RF utilizes bootstrap resampling to select multiple samples from the original dataset to establish a random forest, builds a regression tree for each selected sample, and makes every tree involved in the prediction process. Eventually, all the trees’ regression results are averaged to be the final prediction [36]. In our work, the RF was constructed based on a dataset including environmental factors and a dataset excluding environmental factors separately. The division of the training set and test set was in accordance with LOOCV, where each sample was taken as test set and the remaining samples were considered as the training set, until every sample was taken as test set once. Finally, the RMSE, MAE, and MaxAE of the RF model were computed.

#### 3.3.3. Body Temperature Prediction Model Based on BPNN

BPNN is a multi-layer feed-backward neural network based on error reverse propagate. BPNN is a very adaptive with irregular models. The topology structure of BPNN consists of an input layer, a hidden layer, and an output layer, and the neurons between each layer are connected with weight parameters. The algorithm utilizes the weight parameters within neurons to propagate error between the output layer and hidden layer back and forth, revising the weight parameters and threshold values and minimizing the error [32,37]. In this experiment, the BPNN models were constructed using inputs include environmental factors and inputs without environmental factors. To establish the BPNN models, the tangent sigmoid transfer function (tansig) and the linear transfer function (purelin) were used as the activation functions for the hidden layer and the output layer. In the error back propagation process, the Stochastic Gradient Descent (SGD) was used to minimize the MSE between the BPNN′s output and the ground-truth temperature. The training epoch was 100, and both the expected error and learning rate were set to 0.01. More importantly, the number of hidden layer neurons determines the network structure and prediction performance, which is related to the number of input and output nodes and the complexity of the prediction problem. Insufficient neurons may lead to under-fitting and lower the prediction accuracy, while too many neurons will easily fall into a local minimum or over-fitting. In order to determine the optimal number of neurons in the hidden layer, a total of eight cases of BPNN models were constructed by different numbers of hidden layer neurons, which were set to 2, 4, 6, 8, 10, 12, 14, and 16, respectively. The leave-one-out cross validation method was used to test the model’s performance, and each case was tested 30 times. The test results show that the average MSE along with the hidden layer neurons was first decreased and then increased, and the number of neurons with the minimum MSE was 10. Therefore, it can be concluded that the network structure with 10 neurons in the hidden layer is an appropriate BPNN model for pig temperature prediction.

## 4. Results

**Figure 4:**
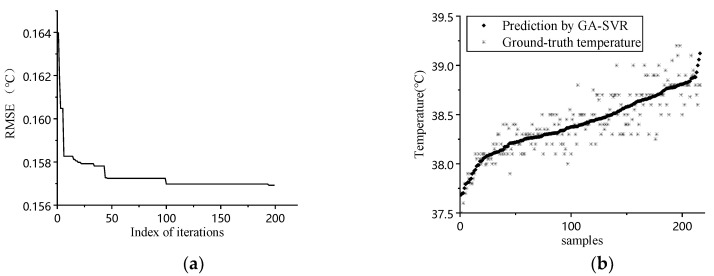

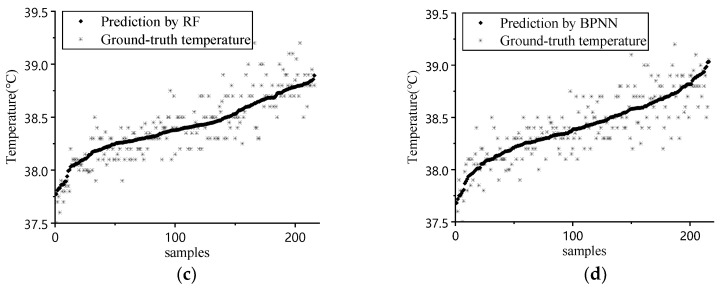
(**a**) Iteration process of GA; (**b**) prediction result comparison of GA-SVR; (**c**) prediction result comparison of RF; (**d**) prediction result comparison of BPNN.

The data obtained from our animal experiment were processed according to the models discussed in Section 3.3, and the result is shown in Figure 4. The Figure 4a shows the result of MSE vs. iteration in GA. Judging from the graph, the fitness increases when iteration progress goes on. The optimum solution for *C*, *g*, and *e* are 0.835, 0.161, and 0.028, respectively. Figure 4b–d shows the prediction results and ground-truth comparison for GA-SVR, RF, and BPNN. 

Judging from the prediction results, the prediction result follows the general trend of ground-truth data when environmental factors were considered. The quantitative analysis for different inputs and models is shown in Table 2.

From the results in Table 2, it is evident that the models incorporating environmental factors yielded a lower MaxAE, MAE, and RMSE than the models that did not consider environmental influences. The MaxAE, MAE, and RMSE for the three models reduced from 0.845, 0.209, and 0.274 °C to 0.568, 0.131, and 0.169 °C after incorporating the environmental factors. The coefficient correlation *r* for the prediction and ground-truth dramatically increased from 0.518 to 0.847, which indicates that the incorporation of environmental factors was necessary for the successful prediction of the pigs′ body temperature. Besides this, comparing the three models incorporating environmental factors, GA-SVR demonstrated the best results, with the MaxAE, MAE, RMSE, and *r* settling at 0.478, 0.124, 0.159, and 0.863 (*p* < 0.01). The RFs were all weaker in terms of four indicators than GA-SVR, and BPNN performed the worst in all respects. In the modes without the consideration of environmental factors, RF demonstrated the best outcome, GA-SVR ranked in the middle, and BPNN was the worst. This comparison shows that RF was the most robust among the three models. In conclusion, GA-SVR performed the best when considering environmental factors for the prediction of pigs′ body temperature, RG was the most robust and stable algorithm, and BPNN was the least reliable for pig body temperature prediction.

## 5. Discussions

This paper investigated into the methodology of utilizing IRT for measuring the core temperature of pigs. Using IRT to take the ROI temperatures of the eye, ear root, backside, and vulva and analyzing the relationship between the surrounding indicators and the ROI temperatures, it is discovered that the ROI temperatures of the ear root, eye, and backside were highly influenced by the surrounding indicators. Our result corresponded to that of Loughmiller [38] and Wendt [39]. Wendt et al. discovered that the correlation coefficient between the ear root temperature and air temperature was 0.26 [36], however Wendt believed that humidity had little influence on skin temperatures, which contradicted our findings. From our analytical results, despite the ROI temperatures of the ear root and vulva having little interaction, their correlation coefficients were −0.21 and −0.25, respectively (*p* < 0.01). Therefore, it was necessary to consider the air temperature and humidity when constructing the prediction model. Our models incorporating environmental factors were one of the highlights, with a significant improvement on the performance results regarding the RMSE, MAE, and R values.

But this research has limitations. The animal experiment was conducted within a week in summer, when the environmental factors tend to be stable, limiting the range of our dataset. The averages of air temperature and relative humidity were 28.97 ± 1.52 °C and 76.09 ± 7.77%, which demonstrated that most air temperature data within our dataset were distributed between 27.45 °C and 30.49 °C, and those of relative humidity were between 68.32% and 83.86%. Our dataset was limited in variation, and thus cannot represent the air temperature and humidity vicissitude year-round, making our model′s application limited to a certain season. Despite the shortcomings, judging from our results, all the prediction models considering environmental factors all performed better than those that did not consider environmental factors. This demonstrated that it is necessary to consider the air temperature and humidity when modeling the core temperature of swine.

Further research should cover a wider range of datasets, with variations in seasons, making the changes in temperature and humidity more dramatic. Further modeling should be based on datasets of a wider range, making the model more applicable across the year. Different setups and arrangements of the pig farm might be susceptible to divergent environmental factors, or under different degrees of influence, and future investigations should adaptively vary environmental factors for specific pig farm circumstances. In terms of the model construction, further investigation into the performance of deep learning algorithms on pig internal temperature prediction is needed. The methods applied in our paper all have no deep layers, and their capability to generalize complicated relationships may be insufficient, and they may fall into the trap of over fitting and local maximum. Deep learning mimics the functions of the human brain or biological neural networks to abstract and model patterns. It is a deep and nonlinear neural structure, and can approximate more complicated functions. Further research should investigate the application of deep leaning in pig temperature prediction.

## 6. Conclusions

In order to improve the accuracy of non-contact IRT in pig internal body temperature measurement, this paper investigated the influence of air temperature and humidity on the skin temperatures of ROIs and incorporated environmental factors into prediction models. The conclusions are as follows:

1. Through analyzing the correlation between the skin temperatures of ROIs from infrared images and environmental factors, it is evident that the backside, eye, and ear root temperature were heavily correlated with the air temperature, with the correlation coefficients at 0.52, 0.32, and 0.28 (*p* < 0.01), respectively. The backside temperature and eye temperature were also influenced by humidity, with the correlation coefficients at −0.21 and −0.25. This indicates that the air temperature and humidity influence ROI temperatures through heat exchange.
2. Judging from the result of the BPNN, RF, and GA-SVR based on the dataset incorporating environmental factors and the dataset without environmental factors, the former dataset yielded the average MaxAE, MAE, and RMSE of 0.556, 0.134, and 0.171 °C, and the highest *r* was 0.837. The latter dataset yielded the average MaxAE, MAE, and RMSE of 0.865, 0.212, and 0.275 °C, and the highest *r* was 0.501. This indicates that incorporating environmental factors is necessary for the successful prediction of pigs′ body temperature.
3. Comparing the three models incorporating environmental factors, GA-SVR exhibited the most superior outcomes regarding the MaxAE, MAE, RMSE, and *r*, reaching 0.478, 0.124, 0.159 °C, and 0.863 (*p* < 0.01). All RF’s evaluation indicators were slightly weaker than those of GA-SVR, and BPNN was the worst in all terms. It is concluded that GA-SVR was the optimum choice regarding the dataset incorporating environmental factors; RF was the most stable and robust, with a relatively high precision; and BPNN was the least suitable. GA-SVR and RF were both applicable for the prediction of pigs′ body temperature, however BPNN is less likely to construct an accountable prediction model for our task.

## Figures and Tables

**Table 1:** Swine ROI temperature statistical characteristics and correlation with environmental factors.

Indicator	Maximum	Minimum	Mean	Standard Error	Air Temperature Correlation	Humidity Correlation
***T_back_* (°C)**	38.73	35.18	36.81	0.73	0.52 **	−0.21 **
***T_vulva_* (°C)**	38.76	33.77	36.95	0.84	0.13	0.07
***T_eye_* (°C)**	38.86	36.24	37.43	0.48	0.32 **	−0.25 **
***T_ear_* (°C)**	39.12	36.10	37.47	0.64	0.28 **	0.03
***T_air_* (°C)**	33.10	26.00	28.97	1.52	—	−0.81 **
***RH_air_* (%)**	91.80	62.00	76.09	7.77	−0.81 **	—

Note: ** represents a significance of *p* < 0.01.

**Table 2:** With and without environmental in modeling comparison results.

	Prediction Result of Models Integrating Environmental Factors				Prediction Result of Models without Integrating Environmental Factors			
Model	MaxAE (°C)	MAE (°C)	RMSE (°C)	*r*	MaxAE (°C)	MAE (°C)	RMSE (°C)	*r*
BPNN	0.656	0.142	0.183	0.823 **	0.934	0.228	0.299	0.458 **
RF	0.569	0.126	0.164	0.856 **	0.745	0.193	0.250	0.616 **
GA-SVR	0.478	0.124	0.159	0.863 **	0.855	0.207	0.272	0.518 **
Mean	0.568	0.131	0.169	0.847	0.845	0.209	0.274	0.518

Note: ** indicates *p* < 0.01.
